# HIV PrEP use and unmet need among gay, bisexual and other men who have sex with men in London: An analysis of community cross‐sectional surveys in England 2019–2022

**DOI:** 10.1111/hiv.70157

**Published:** 2025-11-30

**Authors:** Flavien Coukan, Dana Ogaz, John Saunders, Gary Murphy, Arham Khawar, Iman Scarlett, Hamish Mohammed, Fiona Burns

**Affiliations:** ^1^ Institute for Global Health University College London London UK; ^2^ Blood Safety, Hepatitis, STI and HIV Division UK Health Security Agency London UK; ^3^ Public Health Microbiology Reference Services UK Health Security Agency London UK

**Keywords:** bisexual and other men who have sex with men, gay, health equity, HIV prevention, pre‐exposure prophylaxis, sexual health services

## Abstract

**Objectives:**

In England, HIV pre‐exposure prophylaxis (PrEP) was routinely commissioned at sexual health services from 2020. We compared PrEP use and unmet need among gay, bisexual and other men who have sex with men (GBMSM) in London before (2019) and during (2022) routinely commissioned PrEP and the factors associated with its use.

**Methods:**

Cross‐sectional, self‐administered surveys were conducted in London commercial venues in 2019 (*n* = 1408) and 2022/2023 (*n* = 1090). Anonymous questionnaires collected data on socio‐demographic characteristics, sexual behaviours, service engagement and outcomes. PrEP need was defined as condomless anal sex (CAS) in the last 3 months or with HIV‐positive/unknown status partners not on HIV treatment in the last year. Multivariable logistic regressions examined factors associated with PrEP use.

**Results:**

Among HIV‐negative/unknown GBMSM, current PrEP use more than doubled (19.9% (245/1233) in 2019 to 44.2% (360/814) in 2022 (*p* < 0.001)), representing 2.7‐fold higher odds of PrEP use among GBMSM with identified PrEP need pre‐ to post‐commissioning (aOR: 2.74, 95% CI: 2.13–3.54). Age disparities remained, whereby men aged 40–44 years had higher odds of PrEP use compared to those 18–24 years (aOR: 3.34, 95% CI: 1.93–5.78). Current PrEP users also reported higher healthcare engagement and sexual risk behaviours than those with unmet PrEP need. Meanwhile, unmet PrEP need declined significantly from 67.9% (431/635) in 2019 to 43.8% (212/484) in 2022 (*p*‐value < 0.001).

**Conclusions:**

While routine PrEP commissioning increased PrEP use, age disparities remained, as did high levels of unmet PrEP need among GBMSM in London. This highlights the importance of targeted interventions to achieve equitable PrEP access.

## BACKGROUND

In England, new HIV diagnoses in gay, bisexual and other men who have sex with men (GBMSM) peaked in 2014 and subsequently dropped to a twenty‐year low in 2020 [1]. This decline was driven by the scale‐up of HIV combination prevention in this population through increased condom use, repeat HIV testing, treatment as prevention and limited access to HIV pre‐exposure prophylaxis (PrEP), all of which are key to reaching the government's goal to end new HIV transmissions in England by 2030 [2]. Notably, the fall in new HIV diagnoses among GBMSM occurred in the absence of widespread PrEP availability [3]. However, there has been an increasing trend in bacterial sexually transmitted infections (STIs) since 2014 on a backdrop of outbreaks of mpox and sexually transmitted shigellosis [4], and new HIV diagnoses among GBMSM increased by 18% from 2022 to 2023 [1]. Consequently, GBMSM accounted for 23% of all new HIV diagnoses in 2023 in England, highlighting that this population remains a priority for HIV prevention efforts.

Despite robust evidence that oral and long‐acting injectable PrEP are highly effective at preventing HIV acquisition among GBMSM if taken as prescribed [5, 6, 7, 8], their implementation across the United Kingdom has varied. While Wales and Scotland implemented oral PrEP in 2017, access in England was originally only through the PrEP Impact Trial (October 2017 and July 2020) [9]. This pragmatic health technology assessment expanded from the initial target recruitment numbers of 10 000 to 26 000 due to the rapid uptake of trial places, resulting in lengthy recruitment pauses [10]. Despite expansions, the trial could not keep up with demand, so community‐based organizations facilitated private PrEP purchases for those who could not access it [11]: in a convenience sample of 1742 PrEP users in the United Kingdom, over 30% were buying it from private online providers in 2019. These access limitations potentially influenced both PrEP uptake patterns and sourcing behaviours. Indeed, our previous analysis of the 2019 Gay Men's Sexual Health Survey (GMSHS) revealed that while PrEP use had increased substantially since 2016, considerable unmet need persisted, with over two‐thirds of GBMSM with a known PrEP need not reporting current use at the time [3]. The study also identified concerning disparities in PrEP access, particularly among younger men and those with lower educational attainment [3].

The implementation of routine uncapped PrEP commissioning in sexual health services (SHS) in October 2020 marked a significant shift in the PrEP access landscape in England, theoretically removing many structural barriers to uptake [3]. In 2021, the first full year of PrEP commissioning in England, national surveillance data reported that the number of GBMSM PrEP users more than doubled that of the number of PrEP Impact Trial participants [1]. However, this transition occurred against the backdrop of the COVID‐19 pandemic, which dramatically impacted SHS delivery, potentially affecting PrEP access and SHS use patterns in 2021 [12, 13]. While national surveillance data provide valuable insights into PrEP provision through SHS, community‐based surveillance remains essential for understanding PrEP use and need among GBMSM who may not regularly engage with SHS [14]. This is especially true as the most recent UK clinical guidelines on the use of HIV PrEP move beyond GBMSM‐centric eligibility criteria to include other populations at risk of HIV acquisition (e.g. Black African men and women, transgender women, recent migrants and people who inject drugs) and towards expanded provision in community settings [15, 16]: they recommend that PrEP provision should be commissioned in community pharmacies, drug and alcohol services, primary care and online services.

The GMSHS, a serial, anonymized, cross‐sectional, self‐administered survey in London commercial venues (e.g. clubs, bars, saunas) of GBMSM, provides periodic monitoring of HIV/STI testing, sexual risk behaviours and HIV prevention use. The 2022 round of the GMSHS offers a unique opportunity to examine changes in PrEP use and unmet need following routine implementation and to assess progress towards equitable PrEP access—a crucial element in achieving England's goal of ending HIV transmission by 2030 [2]. This study aims to: (a) compare PrEP use and unmet PrEP need among GBMSM in London before (2019) and during (2022) the period of routine implementation; (b) examine changes in demographic and behavioural factors associated with PrEP use and unmet need; and (c) assess progress toward equitable PrEP access across different subpopulations of GBMSM.

## METHODS

### Study design and setting

Survey methods have been described previously [3, 17, 18], see Supplement S1 for the 2022 survey. Briefly, between June and August 2019 and November 2022 and February 2023, trained fieldworkers visited 34 and 24 London venues and events primarily frequented by GBMSM, respectively. Venues and events were collated from previous survey listings, publications, online forums and anecdotal evidence from community stakeholder feedback. At those participating venues, trained fieldworkers invited all men aged ≥18 to self‐complete a short, self‐completed, anonymized sexual health questionnaire and, if willing, provide an oral fluid sample for anonymous HIV Antibody (Ab) testing.

### Data collection

Participants provided verbal informed consent after reading through the study information sheet before completing the survey. The anonymous questionnaire collected data on socio‐demographic characteristics, sexual behaviours, HIV and STI testing patterns and outcomes, SHS utilization, HIV prevention strategies and drug use before or during sex. Both survey rounds used consistent questions to allow for longitudinal comparison, with the 2022 survey introducing questions about Mpox diagnosis, vaccination and attitudes, which were not used for this analysis.

### Oral fluid collection and HIV Ab testing

After completing the survey, participants were asked to provide an oral saliva sample for anonymous Ab HIV testing using the Intercept i2 device (OraSure Technologies, Bethlehem, PA, USA). Refusal to provide a sample did not prevent participation in the study. Samples were stored anonymously in tamper‐proof envelopes and transported to UK Health Security Agency laboratories for processing within 21 days following collection. There, questionnaires and oral fluid samples were linked using matching barcode stickers to maintain anonymity. Specimens were then tested for total IgG as a quality check and for anti‐HIV‐1/2 by GACELISA (Abbott Laboratories). Reactive specimens underwent further confirmatory testing as previously described [19].

### Definitions and measures

For these analyses, GBMSM were defined as those who self‐identified as men (including transmen) and self‐reported as gay or bisexual or having oral or anal sex with another man in the last year. Men under the age of 18 who completed a questionnaire were excluded from the analysis. HIV status was based on self‐report or, where not specified, on the use of HIV antiretroviral treatment (ART) or the reported result of the last HIV test.

In line with previous methodology and 2018 UK PrEP clinical guidelines [15], PrEP need was defined as reporting either any condomless anal sex (CAS) in the last 3 months, or CAS in the last year with an HIV‐positive /unknown status partner not known to be on ART. This PrEP‐need definition used the more conservative three‐month look‐back window of the PrEP Impact Trial eligibility criteria when the 2019 survey round took place, compared to the six‐month window listed in the 2018 UK PrEP clinical guidelines. Unmet PrEP need was defined as having an identified PrEP need but no reported current PrEP use.

### Statistical analysis

All data were double‐entered using Microsoft Access 2010, and any discordance was resolved by a third reviewer. Data analyses were performed using Stata v18.0 (StataCorp, College Station, TX, USA). Descriptive analyses of sociodemographic characteristics, SHS engagement and outcomes (e.g. number of SHS visits and STI diagnoses in the last year and last HIV test), along with sexual risk and prevention behaviours, were undertaken in all GBMSM and among self‐perceived HIV‐negative /unknown status GBMSM who provided information on current PrEP use.

Multivariable logistic regression was used to investigate the association between current PrEP use and sociodemographic characteristics in GBMSM with a PrEP need, using survey years (i.e. 2019 vs. 2022) as covariates. Bivariate logistic regressions were done to assess if the characteristics should be retained in the multivariable models (*p*‐value < 0.10), and evidence of association was considered significant where *p*‐value < 0.05.

Multivariable logistic regressions investigated the association between current PrEP use and (a) SHS engagement and outcomes and (b) sexual risk and prevention behaviours for the 2022 data. In this stepped approach, variables with bivariate association (*p*‐value < 0.10) were adjusted for the sociodemographic characteristics carried forward from prior models.

Unadjusted odds ratios (ORs), adjusted odds ratios (aORs), 95% confidence intervals (CIs) and associated *p*‐values derived from the likelihood ratio test were calculated. Finally, all analyses were based on available information (no missing data were imputed).

## RESULTS

### Survey sample

A total of 1535 questionnaires were completed in the 2019 survey round compared to 1254 questionnaires in 2022, of which 1408 and 1090 were eligible for analysis, respectively. Similar proportions of those survey participants were classified as HIV‐negative or unknown status: 91.5% (1288/1408) and 93.5% (1019/1090) in 2019 and 2022 (*p*‐value = 0.061), respectively (Table 1). The median age of those HIV‐negative/unknown GBMSM was 35 years (Interquartile range [IQR]: 28–44) in 2019 and 36 years (IQR: 30–46) in 2022. In both survey periods, the majority of HIV‐negative/unknown participants were of white ethnicity (75.5% in 2019 and 72.4% in 2022; *p*‐value = 0.095), lived in London (81.3% in 2019 and 77.7% in 2022; *p*‐value = 0.034) and reported higher educational levels (≥2 years education since age 16 or still in full‐time education: 84.4% in 2019 and 85.3% in 2022; *p*‐value = 0.567).

**TABLE 1:** Comparing sociodemographic characteristics, service engagement and outcomes, sexual risk and prevention behaviours in 2019 and 2022 among (a) HIV‐negative/unknown GBMSM, (b) GBMSM providing information on current PrEP use and (c) GBMSM current pre‐exposure prophylaxis (PrEP) users.

	(a) HIV‐negative/unknown GBMSM^a^, ^b^		(b) GBMSM providing information on current PrEP use^a^, ^b^, ^c^		(c) GBMSM reporting current PrEP use	
	2019	2022	2019	2022	2019	2022
	*n* = 1288	*n* = 1019	*n* = 1233	*n* = 814	*n* = 245	*n* = 360
Recruitment location						
Bar/pub	72.6% (935/1288)	80.4% (819/1019)	72.4% (893/1233)	80.3% (654/814)	70.6% (173/245)	79.7% (287/360)
Club	23.1% (298/1288)	18.7% (190/1019)	23.2% (286/1233)	18.8% (153/814)	24.1% (59/245)	19.4% (70/360)
Sauna	4.3% (55/1288)	1.0% (10/1019)	4.4% (54/1233)	0.9% (7/814)	5.3% (13/245)	0.8% (3/360)
Provided oral fluid sample						
No	38.3% (493/1288)	29.9% (305/1019)	37.5% (462/1233)	29.4.% (239/814)	35.1% (86/245)	24.7% (89/360)
Yes	61.7% (795/1288)	70.1% (714/1019)	62.5% (771/1233)	70.6% (575/814)	64.9% (159/245)	75.3% (271/360)
HIV Ab result^d^						
Indeterminate	0.1% (1/784)	0.6% (4/696)	0.1% (1/760)	0.7% (4/562)	0.7% (1/155)	0.7% (2/270)
Negative	99.1% (777/784)	98.4% (685/696)	99.1% (753/760)	98.2% (552/562)	97.4% (151/155)	98.9% (267/270)
Positive	0.8% (6/784)	1.0% (7/696)	0.8% (6/760)	1.1% (6/562)	2.0% (3/155)	0.4% (1/270)
Sociodemographic characteristics						
Age						
Mean [SD]	37 [11.1]	39 [11.7]	37 [11.1]	38 [11.5]	35 [9.2]	37 [10.1]
Median [IQR]	35 [28–44]	36 [30–46]	35 [28–44]	35 [30–45]	33 [28–41]	35 [30–42]
Age group						
18–24	10.8% (138/1276)	6.6% (66/995)	10.8% (132/1223)	7.4% (59/796)	8.6% (21/244)	5.7% (20/352)
25–34	39.1% (499/1276)	38.8% (386/995)	38.5% (471/1223)	40.1% (319/796)	43.9% (107/244)	44.0% (155/352)
35–44	27.3% (348/1276)	26.4% (263/995)	27.6% (337/1223)	26.6% (212/796)	35.3% (86/244)	29.6% (104/352)
≥45	22.8% (291/1276)	28.1% (280/995)	23.1% (283/1223)	25.9% (206/796)	12.3% (30/244)	20.7% (73/352)
Ethnic group						
White	75.5% (971/1286)	72.4% (736/1016)	75.6% (930/1231)	72.4% (587/811)	72.2% (177/245)	72.4% (260/359)
Black	3.9% (50/1286)	3.0% (30/1016)	3.9% (48/1231)	2.7% (22/811)	3.3% (8/245)	3.1% (11/359)
South East Asian	2.4% (31/1286)	2.8% (28/1016)	2.3% (28/1231)	2.8% (23/811)	2.9% (7/245)	3.1% (11/359)
Asian	4.6% (59/1286)	4.2% (43/1016)	4.6% (57/1231)	4.4% (36/811)	5.3% (13/245)	3.9% (14/359)
Latin American	4.4% (57/1286)	9.1% (92/1016)	4.5% (55/1231)	8.6% (70/811)	5.3% (13/245)	9.2% (33/359)
Mixed/other	9.2% (118/1286)	8.6% (87/1016)	9.2% (113/1231)	9.0% (73/811)	11.0% (27/245)	8.4% (30/359)
UK‐born						
No	46.4% (589/1269)	44.1% (443/1005)	46.1% (560/1216)	44.3% (356/803)	49.4% (119/241)	47.2% (167/354)
Yes	53.6% (680/1269)	55.9% (562/1005)	54.0% (656/1216)	55.7% (447/803)	50.6% (122/241)	52.8% (187/354)
Residence						
London	81.3% (1026/1262)	77.7% (791/1018)	81.4% (983/1208)	79.3% (645/813)	85.4% (204/239)	83.3% (300/360)
Outside London	12.1% (153/1262)	13.0% (132/1018)	12.2% (147/1208)	11.8% (96/813)	8.4% (20/239)	9.7% (35/360)
Outside UK	6.6% (83/1262)	9.3% (95/1018)	6.5% (78/1208)	8.9% (72/813)	6.3% (15/239)	6.9% (25/360)
Current employment						
No	12.2% (155/1274)	10.0% (99/994)	12.0% (146/1221)	9.3% (74/794)	9.9% (24/243)	7.9% (28/353)
Yes	87.8% (1119/1274)	90.0% (895/994)	88.0% (1075/1221)	90.7% (720/794)	90.1% (219/243)	92.1% (325/353)
Education since age 16						
0–2 years	15.6% (196/1260)	14.7% (147/1001)	15.6% (188/1208)	14.3% (115/803)	11.2% (27/241)	13.5% (48/356)
≥2 years/still full‐time	84.4% (1064/1260)	85.3% (854/1001)	84.4% (1020/1208)	85.7% (688/803)	88.8% (214/241)	86.5% (308/356)
Service engagement and outcomes						
SHS visit in the last year						
No	32.3% (409/1267)	35.8% (357/997)	32.5% (395/1217)	29.4% (235/800)	2.9% (7/243)	2.8% (10/355)
Yes	67.7% (858/1267)	64.2% (640/997)	67.5% (822/1217)	70.6% (565/800)	97.1% (236/243)	97.2% (345/355)
Last HIV test						
Last 3 months	47.0% (597/1269)	40.9% (400/979)	47.5% (578/1217)	46.5% (367/789)	88.1% (214/243)	78.1% (274/351)
Between 3 and 12 months	25.7% (326/1269)	25.8% (253/979)	25.2% (307/1217)	26.7% (211/789)	10.7% (26/243)	19.4% (68/351)
More than a year ago	17.8% (226/1269)	19.8% (194/979)	17.9% (218/1217)	16.6% (131/789)	0.8% (2/243)	2.3% (8/351)
Over 5 years ago	5.0% (63/1269)	8.0% (78/979)	4.9% (60/1217)	5.1% (40/789)	0.4% (1/243)	0.0% (0/351)
Never	4.5% (57/1269)	5.5% (54/979)	4.4% (54/1217)	5.1% (40/789)	0.0% (0/243)	0.3% (1/351)
≥2 HIV tests in the last year						
No	45.2% (528/1168)	51.3% (501/976)	45.0% (503/1119)	44.3% (348/786)	6.7% (16/239)	11.5% (40/349)
Yes	54.8% (640/1168)	48.7% (475/976)	55.1% (616/1119)	55.7% (438/786)	93.3% (223/239)	88.5% (309/349)
≥4 STI tests in the last year						
No	82.0% (956/1166)	83.6% (811/970)	81.8% (921/1126)	81.0% (632/780)	50.6% (119/235)	64.2% (224/349)
Yes	18.0% (210/1166)	16.4% (159/970)	18.2% (205/1126)	19.0% (148/780)	49.4% (116/235)	35.8% (125/349)
STI diagnosis in the last year						
No	76.4% (960/1256)	75.7% (733/968)	76.4% (922/1207)	71.9% (562/782)	41.0% (100/244)	53.3% (188/353)
Yes	23.6% (296/1256)	24.3% (235/968)	23.6% (285/1207)	28.1% (220/782)	59.0% (144/244)	46.7% (165/353)
Sexual risk and prevention behaviours						
CAS in the last 3 months						
No	49.5% (625/1262)	42.0% (415/988)	49.6% (603/1216)	39.3% (313/797)	16.9% (41/243)	23.0% (81/353)
Yes	50.5% (637/1262)	58.0% (573/988)	50.4% (613/1216)	60.7% (484/797)	83.1% (202/243)	77.1% (272/353)
≥5 CAS partners in the last year						
No	79.4% (904/1138)	72.8% (638/877)	79.1% (867/1096)	69.0% (501/726)	39.4% (93/236)	40.4% (130/322)
Yes	20.6% (234/1138)	27.3% (239/877)	20.9% (229/1096)	31.0% (225/726)	60.6% (143/236)	59.6% (192/322)
Chemsex in the last year^e^						
No	83.7% (1025/1225)	85.2% (795/933)	83.8% (990/1182)	82.7% (623/753)	66.0% (155/235)	75.8% (257/339)
Yes	16.3% (200/1225)	14.8% (138/933)	16.2% (192/1182)	17.3% (130/753)	34.0% (80/235)	24.2% (82/339)
HIV PEP in the last year						
No	93.1% (1199/1288)	91.0% (927/1019)	93.2% (1149/1233)	89.1% (725/814)	84.1% (206/245)	81.7% (294/360)
Yes	6.9% (89/1288)	9.0% (92/1019)	6.8% (84/1233)	10.9% (89/814)	15.9% (39/245)	18.3% (66/360)
DoxyPEP^f^						
No	94.9% (1154/1216)	86.6% (817/943)	95.0% (1109/1168)	84.0% (637/758)	87.3% (207/237)	72.0% (239/332)
Yes	5.1% (62/1216)	13.4% (126/943)	5.1% (59/1168)	16.0% (121/758)	12.7% (30/237)	28.0% (93/332)
PrEP use in the last year						
No	75.5% (925/1225)	53.7% (539/1004)	75.5% (923/1223)	43.9% (357/813)	0.0% (0/245)	0.0% (0/360)
Yes	24.5% (300/1225)	46.3% (465/1004)	24.5% (300/1223)	56.1% (456/813)	100% (245/245)	100% (360/360)

^a^ Self‐identified men, including trans men, who self‐reported as gay or bisexual, or who had sex with a man in the last year and did not previously participate in the survey in the last 3 months.

^b^ Based on self‐perceived HIV status; where self‐reported HIV status not specified, based on report of last HIV test as positive or antiretroviral medication use.

^c^ Self‐perceived HIV‐negative/unknown GBMSM.

^d^ Where IgG >0.200.

^e^ Chemsex defined as self‐reported use of ketamine, gamma hydroxybutyrate(GHB)/gamma butyrolactone(GBL), mephedrone and/or meth amphetamine before or during sex.

^f^ Doxycycline post‐exposure prophylaxis (doxy PEP) to prevent bacterial STIs.

### HIV Ab testing

Oral fluid samples with suitable immunoglobulin G (IgG) levels for HIV Ab testing were provided by 60.7% (855/1408) and 68.9% (751/1090) of HIV‐negative/unknown participants in 2019 and 2022, respectively (Supplement S2; *p*‐value<0.001). Of those samples, 8.3% (71/855) and 7.5% (56/751) were HIV Ab positive in 2019 and 2022, respectively (*p*‐value = 0.530). Furthermore, when looking at those who were HIV Ab positive, 8.5% (6/71) of GBMSM self‐reported as HIV‐negative in 2019 and 12.5% (7/56) in 2022 (*p*‐value = 0.559); this resulted in a 0.8% (6/784; 95% CI: 0.3%–1.7%) and 1.0% (7/696; 95% CI: 0.4%–2.1%) undiagnosed HIV prevalence (*p*‐value = 0.782) among HIV‐negative/unknown self‐perceived GBMSM who provided a suitable sample (Table 1).

### PrEP use in the last year

**FIGURE 1:**
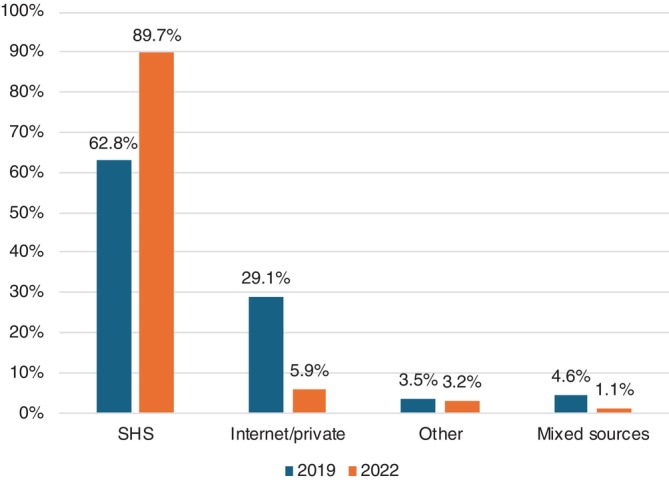
Pre‐exposure prophylaxis (PrEP) sourcing in self‐perceived HIV‐negative/unknown MSM reporting PrEP use in the last year.

PrEP use in the last year among HIV‐negative/unknown GBMSM nearly doubled from 24.5% (300/1225) in 2019 to 46.3% (465/1004) in 2022 (*p*‐value<0.001) (Table 1). While SHS accounted for the largest source of PrEP prescription across survey rounds (Figure 1), its share increased from 62.8% in 2019 (164/261) to 89.7% (393/438) in 2022 (*p*‐value<0.001). This is in contrast with internet/private PrEP prescriptions, which accounted for a significantly smaller proportion of PrEP users over time, from 29.1% (76/261) to 5.9% (26/438) in 2019 and 2022, respectively (*p*‐value<0.001).

### Sociodemographic patterns in PrEP use and unmet PrEP need

Similarly to PrEP use in the last year, current PrEP use more than doubled from 19.9% (245/1233) of self‐perceived HIV‐negative/unknown GBMSM in 2019 to 44.2% (360/814) in 2022 (*p*‐value<0.001) (Table 1). In the multivariate analysis, GBMSM with identified PrEP need in 2022 were 2.7‐fold more likely to report current PrEP use compared to those surveyed in 2019 after adjusting for age group and employment status (aOR: 2.74, 95% CI: 2.13–3.54) (Table 2). Conversely, unmet PrEP need dropped from 67.9% (431/635) in 2019 to 43.8% (212/484) in 2022 (*p*‐value<0.001).

**TABLE 2:** Select sociodemographic characteristics associated with self‐reported current PrEP use in self‐perceived HIV‐negative/unknown GBMSM with identified PrEP need, 2019 and 2022 survey iterations.

	GBMSM with identified PrEP‐need		OR (95% CI)	*p* value	aOR^a^ (95% CI)	*p* value
	Current PrEP use *n* = 476	No current PrEP use *n* = 643				
Survey year						
2019	42.9% (204/476)	67.0% (431/643)	1 (ref)	<0.001	1 (ref)	<0.001
2022	57.1% (272/476)	33.0% (212/643)	2.71 (2.12–3.46)		2.74 (2.13–3.54)	
Age‐group (years)						
18–24	7.2% (34/472)	13.7% (87/636)	1 (ref)	<0.001	1 (ref)	<0.001
25–29	21.4% (101/472)	21.1% (134/636)	1.93 (1.20–3.10)		1.99 (1.20–3.29)	
30–34	24.2% (114/472)	22.8% (145/636)	2.01 (1.26–3.21)		1.90 (1.16–3.12)	
35–39	17.0% (80/472)	13.2% (84/636)	2.44 (1.48–4.02)		2.21 (1.30–3.76)	
40–44	15.7% (74/472)	9.9% (63/636)	3.01 (1.79–5.05)		3.34 (1.93–5.78)	
≥45	14.6% (69/472)	19.3% (123/636)	1.44 (0.88–2.35)		1.46 (0.87–2.46)	
Ethnic group						
White	74.6% (355/476)	74.6% (479/642)	1 (ref)	0.991	‐	‐
Ethnic minority	25.4% (121/476)	25.4% (163/642)	1.00 (0.76–1.32)			
UK‐born						
No	50.0% (234/468)	46.0% (293/637)	1 (ref)	0.188	‐	‐
Yes	50.0% (234/468)	54.0% (344/637)	0.85 (0.67–1.08)			
UK resident						
No	6.8% (32/472)	7.6% (48/632)	1 (ref)	0.604	‐	‐
Yes	93.2% (440/472)	92.4% (584/632)	1.13 (0.71–1.80)			
Current employment						
No	7.2% (34/471)	11.4% (73/640)	1 (ref)	0.018	1 (ref)	0.1118
Yes	92.8% (437/471)	88.6% (567/640)	1.65 (1.08–2.53)		1.43 (0.91–2.25)	
Education since age 16						
None/up to 2 years	12.7% (60/474)	16.1% (102/633)	1 (ref)	0.106	‐	‐
≥2 years/still full‐time	87.3% (414/474)	83.9% (531/633)	1.33 (0.94–1.87)			

^a^ Adjusted for year of survey iteration, age‐group and employment.

Among current PrEP users, the median age increased slightly from 33 years (IQR: 28–41) in 2019 to 35 years (IQR: 30–42) in 2022 (*p*‐value = 0.600), while the distribution by ethnicity remained stable (Table 1): those of white ethnicity accounted for the largest ethnicity grouping across survey rounds (72.2% in 2019 and 72.4% in 2022, *p*‐value = 0.962). Employment among PrEP users remained high between surveys, at 90.1% and 92.1% in 2019 and 2022, respectively (*p*‐value = 0.408). The proportion of GBMSM PrEP users reporting CAS in the last 3 months persisted at around four in five users (83.1% in 2019 and 77.1% in 2022, *p*‐value = 0.071). However, the proportion of PrEP users reporting an STI diagnosis in the last year dropped significantly (Table 1): 59.0% (144/244) reported an STI diagnosis in 2019 and 46.7% (165/353) in 2022 (*p*‐value = 0.003).

The multivariate regression showed significant age‐related disparities in PrEP use: GBMSM aged 40–44 years were over three times more likely to use PrEP when they completed the survey compared to the youngest group (18–24 years [aOR: 3.34, 95% CI: 1.93–5.78]), the highest odds across any age grouping (Table 2). Meanwhile, the youngest GBMSM [18, 19, 20, 21, 22, 23, 24] were some of the least likely to be using PrEP, along with those >45 years. Although significant in the bivariate logistic regression, employment status was only marginally associated with current PrEP use in the multivariate model adjusted for survey year and age (aOR: 1.43, 95% CI: 0.91–2.25). Ethnicity, UK residence and birth and education status were not significant in the bivariate logistic regressions and therefore not included in the adjusted model.

### Healthcare engagement and sexual risk and prevention behaviours

Current PrEP users reported significantly higher healthcare engagement than HIV‐negative/unknown GBMSM who met our PrEP eligibility criteria but who did not use PrEP (i.e. unmet PrEP need) in 2022: after adjusting for age, current PrEP users were over 20 times more likely to have had ≥2 HIV tests in the last year (aOR: 20.47, 95% CI: 12.08–34.69; Table 3) and were two times more likely to have had their last HIV test at an SHS than self‐sampling/testing (aOR: 0.50, 95% CI: 0.32–0.78) compared to those with unmet PrEP need. Additionally, current PrEP users were nearly five times more likely to have had an STI diagnosis in the last year than non‐users with a PrEP need (aOR: 4.75, 95% CI: 3.08–7.31).

**TABLE 3:** Service engagement and outcomes, and sexual risk and prevention behaviours associated with self‐reported current PrEP use in self‐perceived HIV‐negative/unknown GBMSM with identified PrEP need, November 2022 to February 2023.

	GBMSM with identified PrEP‐need		OR (95% CI)	*p* value	aOR^a^ (95% CI)	*p* value
	Current PrEP use *n* = 272	No current PrEP use *n* = 212				
Service engagement and outcomes						
SHS visit in the last year						
No	2.2% (6/271)	45.5% (95/209)	1 (ref)	<0.001	1 (ref)	<0.001
Yes	97.8% (265/271)	54.6% (114/209)	36.81 (15.67–86.43)		37.51 (15.84–88.82)	
≥2 HIV tests in the last year						
No	9.4% (25/267)	65.4% (136/208)	1 (ref)	<0.001	1 (ref)	<0.001
Yes	90.6% (242/267)	34.6% (72/208)	18.28 (11.08–30.18)		20.47 (12.08–34.69)	
Location of last HIV test						
SHS	77.7% (209/269)	59.9% (121/202)	1 (ref)	<0.001	1 (ref)	<0.001
Self‐sampling/ testing	19.0% (51/269)	27.7% (56/202)	0.53 (0.34–0.82)		0.50 (0.32–0.78)	
Other	3.4% (9/269)	12.4% (25/202)	0.21 (0.09–0.46)		0.18 (0.08–0.41)	
STI diagnosis in the last year						
No	47.8% (130/272)	81.4% (166/204)	1 (ref)	<0.001	1 (ref)	<0.001
Yes	52.2% (142/272)	18.6% (38/204)	4.77 (3.12–7.30)		4.75 (3.08–7.31)	
Sexual risk and prevention behaviours						
≥5 CAS partners in the last year						
No	28.3% (69/244)	85.0% (164/193)	1 (ref)	<0.001	1 (ref)	<0.001
Yes	71.7% (175/244)	15.0% (29/193)	14.34 (8.85–23.25)		15.22 (9.22–25.12)	
≥2 casual CAS partners in the last year						
No	26.5% (52/196)	79.3% (138/174)	1 (ref)	<0.001	1 (ref)	<0.001
Yes	73.5% (144/196)	20.7% (36/174)	10.62 (6.54–17.24)		11.21 (6.73–18.69)	
Chemsex in the last year						
No	73.1% (190/260)	83.8% (165/197)	1 (ref)	0.007	1 (ref)	0.007
Yes	26.9% (70/260)	16.2% (32/197)	1.90 (1.19–3.03)		1.92 (1.19–3.08)	
DoxyPEP						
No	69.8% (176/252)	92.4% (182/197)	1 (ref)	<0.001	1 (ref)	<0.001
Yes	30.2% (76/252)	7.6% (15/197)	5.24 (2.90–9.46)		5.52 (3.01–10.09)	
HIV PEP use in the last year						
No	83.1% (226/272)	93.9% (199/212)	1 (ref)	<0.001	1 (ref)	<0.001
Yes	16.9% (46/272)	6.1% (13/212)	3.12 (1.64–5.94)		3.24 (1.68–6.25)	

^a^ Adjusted for age‐group (employment not used as not significant in multivariate model of sociodemographic characteristics).

Furthermore, GBMSM with identified PrEP need who were current PrEP users in 2022 reported higher risk behaviours than those who were non‐users: after adjusting for age, current PrEP users were over 15 times more likely to have had ≥5 CAS partners in the last year than non‐users (aOR: 15.22, 95% CI: 9.22–25.12, Table 3) and were nearly twice as likely to have engaged in chemsex in the last year (aOR: 1.92, 95% CI: 1.19–3.08). However, PrEP users were more likely to report prevention behaviours, including using doxycycline post‐exposure prophylaxis (DoxyPEP) to prevent bacterial STIs (aOR: 5.52, 95% CI: 3.01–10.09) and HIV post‐exposure prophylaxis (PEP) (aOR: 3.24, 95% CI: 1.68–6.25) in the last year.

While the proportion of GBMSM with unmet PrEP need dropped significantly between survey rounds (see above), there were important gaps in prevention coverage in 2022. Only half of GBMSM with unmet PrEP need reported visiting an SHS in the last year (54.6% [114/209]), and nearly one in five (18.6% [38/204]) had an STI diagnosis in the year prior (Table 3). Furthermore, 20.7% (36/174) of GBMSM with unmet PrEP need reported ≥2 casual CAS partners in the last year.

## DISCUSSION

Our findings demonstrate important changes to the PrEP landscape among GBMSM attending London venues following routine PrEP implementation in England, with more than a two‐fold increase in current PrEP use and a significant drop in unmet PrEP need between 2019 and 2022. Despite these improvements, persistent disparities in PrEP use across different demographics indicate that further targeted interventions are needed to achieve equitable PrEP use among GBMSM, especially as sex between men accounts for a disproportionately high number of new HIV diagnoses [1]. Indeed, 43.8% of HIV‐negative/unknown GBMSM who met our conservative definition of PrEP eligibility criteria were not using PrEP.

The increase in current PrEP use from 19.9% in 2019 to 44.2% in 2022 reflects the complex interplay of multiple factors that have shaped the PrEP landscape in England. While the transition from the limited PrEP Impact Trial to uncapped routine implementation of PrEP in SHS provided crucial structural improvements by removing access barriers and increasing availability, this represents one aspect of broader changes in PrEP provision. Another important shift that has occurred between surveys is the growing community awareness and acceptance of PrEP, which was facilitated by health promotion, community‐led advocacy and educational initiatives [20, 21]. Furthermore, after years of familiarization, it is likely that clinicians involved in PrEP provision have adopted a lower threshold for PrEP offers when assessing eligibility. The new BASHH/BHIVA guidelines reflect this shift, emphasizing expanded provision and moving away from restrictive eligibility criteria [16].

The observed increase in PrEP use likely reflects changes in structural availability, community knowledge and acceptance and clinical practice. The corresponding drop in unmet PrEP need from 67.9% in 2019 to 43.8% in 2022 further confirms the combined effect of these multilevel changes in improving PrEP access for GBMSM. The significant shift in PrEP sourcing patterns (whereby SHS accounted for 89.7% of PrEP prescriptions in 2022) further illustrates how the interplay of these factors successfully integrated PrEP into routine SHS care pathways and improved the monitoring of PrEP provision.

Our findings align with previous research on PrEP implementation in the United Kingdom and internationally. The observed increase in PrEP use in England mirrors similar trends in regions that have removed structural barriers to PrEP access [22, 23]. Indeed, the PrEP Impact Trial reported that the initial capacity limitations constrained access, particularly in London, and our data highlight how uncapped implementation has subsequently expanded uptake.

Despite these improvements, disparities in PrEP access remained: our findings showed that younger GBMSM (18 to 24 years) with PrEP need were less likely to use PrEP compared to those 25 to 44, with men aged 40–44 having the highest odds of current PrEP use. This age‐related disparity is consistent with previous research [3, 11, 24]: one qualitative study conducted in 2016 identified specific barriers to PrEP for younger GBMSM, including concerns of stigma, lack of PrEP knowledge and challenges navigating the healthcare system [25]. Inadequate PrEP coverage in younger GBMSM is a concern, especially as they may be at particularly high risk of HIV acquisition [25].

We also observed significant associations between current PrEP use and healthcare engagement, sexual risk behaviours and prevention strategies, which provide important insights into PrEP users. Current PrEP users reported odds 20 times higher of having at least 2 HIV tests in the last year compared to non‐users, likely reflecting the requirement for frequent HIV testing in the PrEP clinical guidelines [15]. Similarly, the higher odds of an STI diagnosis in the last year may also reflect more frequent STI testing (although we cannot distinguish between diagnoses from routine screening vs. symptomatic presentations), which would be consistent with the recommended quarterly frequency of STI testing [15]. It may also be due to higher sexual risk, as evidenced by the higher number of CAS partners reported by PrEP users [5, 26, 27, 28, 29]. However, this analysis could not ascertain whether that is due to GBMSM increasing their sexual risk following PrEP initiation (i.e. risk compensation) or to those already practising sexual risk behaviours being more motivated to seek and more likely to be offered PrEP. Our results show that PrEP users were also more likely to adopt other prevention measures, including doxyPEP to prevent bacterial STIs and HIV PEP. It should be noted that these data were collected prior to the publication of the BASHH guidelines for the use of DoxyPEP [30]. The observed patterns of doxyPEP use among PrEP users also align with emerging research on bacterial STI prevention strategies and highlight the importance of holistic approaches to sexual health [31, 32], although continued monitoring of antimicrobial resistance will be important as DoxyPEP use expands [30].

### Study limitations

Several limitations should be taken into consideration when interpreting our findings. First, the cross‐sectional design limits our ability to establish a causal relationship between reported behaviours and PrEP use. Compared to our cross‐sectional design, longitudinal studies would provide greater insights into the temporal relationship between PrEP initiation and changes in sexual behaviours on healthcare engagement.

Second, our venue‐based sampling strategy may not be representative of the wider London GBMSM population, as it systematically excludes GBMSM who do not frequent commercial venues in London. Men attending these venues may differ from the broader GBMSM population: this is especially true as many included venues host HIV prevention outreach activities, whereby clients may be more aware of HIV prevention strategies like PrEP.

Third, the COVID‐19 pandemic significantly disrupted SHS delivery between the two survey rounds [12, 13], potentially affecting both PrEP access patterns and sexual behaviours. While our study captures the net effect of growing community awareness, routine PrEP implementation and COVID‐19 disruptions, disentangling these is challenging.

Fourth, the data collection relied on self‐reported measures in relation to a lookback period, which could result in recall bias. Furthermore, social desirability bias may also have affected self‐reporting, especially considering the experiences of stigma experienced by PrEP users [33]. However, the anonymous nature of the survey should have minimized the social desirability bias.

Finally, our definition for PrEP needs to use a conservative three‐month look‐back window for CAS, which may underestimate PrEP need compared to the six‐month window used until recently in clinical guidelines [15]. This approach was chosen to maintain consistency with the 2019 GMSHS and the PrEP Impact Trial eligibility criteria in place at the time [34]. However, the updated guideline does not list any look‐back window when assessing PrEP eligibility [16], which should broaden the definition of PrEP need in the future.

Despite these limitations, our study provides valuable insights into the changes to PrEP use and unmet PrEP need following routine implementation in England. This provides important community‐based monitoring to complement national surveillance statistics.

## CONCLUSIONS

The integration of PrEP provision within routine SHS added another tool to the HIV prevention strategy in England and resulted in significant uptake among GBMSM in London. However, substantial gaps in prevention coverage remained, from age‐related disparities to persistent levels of unmet PrEP need. These findings highlight the importance of targeted interventions to achieve equitable PrEP access across all demographic groups, as England works toward its goal of ending HIV transmission by 2030.

## AUTHOR CONTRIBUTIONS

FB, GM, HM, JS, DO and FC designed the study. FB acquired funding. FB, DO, HM, JS and FC reviewed and updated study questionnaires and participant materials. FC managed venue and fieldworker recruitment, training and data collection. GM, AK and IS conceived the laboratory process and provided laboratory interpretation; IS and AK managed specimen processing and performed laboratory testing and validation. FC conducted analyses and wrote the first report draft, with contributions from all authors in successive drafts. All authors reviewed and approved the final report.

## FUNDING INFORMATION

Funding was provided by Gilead Sciences Ltd. and Healthy London Partnership (the Fast Track Cities initiative). FC has received fees for participation in scientific advisory boards from ViiV. FB has received institutional grants from Gilead Sciences Ltd., Viiv Healthcare & MSD, and personal fees form Gilead Sciences Ltd.

## CONFLICT OF INTEREST STATEMENT

FC has received fees for participation in scientific advisory boards from ViiV. FB has received institutional grants from Gilead Sciences Ltd., Viiv Healthcare and MSD, and personal fees from Gilead Sciences Ltd.

## ETHICS STATEMENT

Ethical approval for this study was provided by the London Harrow Research Ethics Committee (REC reference 00/0158). All methods were performed in accordance with relevant guidelines and regulations. Participant approval consent: Verbal informed consent was obtained from all participants.

## Supporting information


**Supplementary S1** Gay Men's Sexual Health Survey (GMSHS) 2022.

**Supplementary S2** Sociodemographic characteristics, service engagement and outcomes, sexual risk and prevention behaviours in GBMSM by self‐perceived HIV status and current PrEP use, 2019 survey iteration.

## Data Availability

Study data from this analysis is available with support from the study sponsor and with a data sharing agreement in place. Requests can be directed to the corresponding author.
