# Supplementary material for: HIV PrEP use and unmet need among gay, bisexual and other men who have sex with men in London: An analysis of community cross‐sectional surveys in England 2019–2022
Source: HIV Med. 2025 Nov 30;27(3):420–32. doi: 10.1111/hiv.70157 (PMC12968497; doi:10.1111/hiv.70157)
Supplement: Supplementary file 1 — Supplementary S1 Gay Men's Sexual Health Survey (GMSHS) 2022. [file HIV-27-420-s002.pdf]

1. Have you already completed this questionnaire in the last three months?

❑ Yes (end here, thank you) ❑ No

2. What was your age at your last birthday?

……………………………… (please specify)

3. In which country were you born?

……………………………… (please specify)

4. Would you describe yourself as Hispanic or Latino?

❑ Yes ❑ No

4a. Which of the following ethnic groups best describes you?

❑ White (British/Irish/Other)
❑ Black (African/Caribbean/British/Other)
❑ South East Asian
❑ Asian (Indian/Pakistani/Bangladeshi)
❑ Mixed
❑ Other……………………………………………………………………

5. Which of the following best describes how you think about yourself?

❑ Man (including trans man) ❑ Non-binary
❑ Woman (including trans woman)
❑ Other……………………………………………………………………

6. What sex were you assigned at birth?

❑ Male ❑ Female
❑ Intersex ❑ Prefer not to say

7. How would you describe your sexual orientation?

❑ Gay/homosexual ❑ Bisexual
❑ Straight/heterosexual
❑ Other……………………………………………………………………

8. Do you currently live in London?

❑ Yes
(Borough/council)………………………………
❑ No – but I live elsewhere in the UK
❑ No – I live outside of the UK
(Country)…………………………………………………………

9. Are you in paid employment at present?

❑ Yes ❑ No

10. How many years of full-time education have you had since you were 16?

❑ None ❑ 2 years or more
❑ Up to 2 years ❑ Still in full-time education

11. In the last year, have you used a sexual health service?

❑ Yes ❑ No **(skip to Q12)**

11a. If yes, in what way(s)? (tick all that apply)

❑ In-person ❑ Online ❑ Over phone

12. What do you believe to be your current HIV status?

❑ Negative ❑ Positive ❑ Don't know

13. When did you have your last HIV test?

❑ Less than 3 months ago
❑ Between 3 and 12 months ago
❑ More than a year ago **(skip to Q15)**
❑ Over 5 years ago **(skip to Q15)**
❑ Never had an HIV test **(skip to Q17)**

14. In the last year, how many HIV tests have you had?

………………… (please specify)

15. Where did you have your last HIV test?

❑ Sexual Health Clinic (in-person)
❑ GP (in-person)
❑ Self-sampling service (sent sample to lab)
❑ Self-testing (immediate result)
❑ Other (please specify)…………………………………

16. What was the result of your last HIV test?

❑ Negative **(skip to Q17)** ❑ Positive
❑ Don't know/did not collect result **(skip to Q17)**

16a. If you are HIV positive, are you currently on antiretroviral medication?

❑ Yes ❑ No

17. In the last year, how many STI tests, excluding HIV, have you had?

………………… (please specify) ❑ None

18. Where did you have your last STI test?

❑ Sexual Health Clinic (in-person)
❑ GP (in-person)
❑ Self-sampling service (sent sample to lab)
❑ Other (please specify)…………………………………
❑ I have never had an STI test

19. In the last year, have you had an STI?

❑ Yes ❑ No ❑ Don't know

20. In the last year, have you been diagnosed with any of the following? (tick all that apply)

❑ Gonorrhoea ❑ Syphilis ❑ Chlamydia
❑ Shigella ❑ LGV ❑ Monkeypox
❑ An STI not listed (please specify)……………………..

21. Have you been offered or sought a monkeypox vaccine?

❑ Yes, I've received the vaccine (**skip to Q22**)
❑ Yes, but vaccine not available (**skip to Q22**)
❑ Yes, but I declined (**skip to Q22**)
❑ No

21a. If no, would you accept a monkeypox vaccine if offered?

❑ Yes ❑ No ❑ Don't know

22. In the last year, have you used antibiotics before or after sex to prevent STIs?

❑ Yes ❑ No ❑ I'm not sure what this is

23. In the last year, how many men have you had sex with (oral or anal)?

………………… (please specify) ❑ None

24. In the last year, how many men have you had anal sex with? ………………… (please specify) ❑ None

25. When was the last time you had anal sex with a man without a condom?

❑ Less than 3 months ago
❑ Between 3 and 12 months ago
❑ More than a year ago **(skip to Q27)**
❑ Never **(skip to Q27)**

26. In the last year, with how many men have you had anal sex without a condom?

.... (please specify) ❑ None (**skip to Q27**)

Of these:

26a. How many were only once?

.... (please specify) ❑ None ❑ Don't know

26b. How many were on PrEP?

.... (please specify) ❑ None ❑ Don't know

26c. How many were HIV positive?

.... (please specify) ❑ None (**skip to Q27**) ❑ Don't know

26d. If HIV positive, how many were on HIV treatment?

.... (please specify) ❑ None ❑ Don't know

27. In the last year, have you used any of the following drugs just before or during sex? (tick all that apply)

❑ Amphetamine (speed)
❑ Cocaine
❑ Meth amphetamine (Crystal meth, ice/glass, Tina)
❑ Ketamine (Vitamin K, K, Special K)
❑ GHB/GBL (Gina, Liquid Ecstasy, Liquid G)
❑ Mephedrone (Drone, MCAT, meow meow)
❑ Poppers (Amyl or other nitrate inhalants)
❑ Other (please specify)...
❑ I have not used any of these drugs (**skip to Q28**)

27a. In the last year, did you inject or slam any of these drugs?

❑ Yes ❑ No

28. In the last year, have you taken antiretroviral drugs after you had sex (i.e. PEP) to reduce the risk of HIV?

❑ Yes ❑ No

29. In the last year, have you taken HIV pre-exposure prophylaxis (i.e. PrEP) to reduce the risk of HIV?

❑ Yes ❑ No (**skip to Q33**)

30. In the last 4 weeks, have you taken PrEP?

❑ Yes ❑ No

31. In the last year, where did you get PrEP? (tick all that apply)

❑ Sexual Health Service (online or in-person)
❑ Internet ❑ Private prescription
❑ Other (please specify)...

32. How do you, or did you, last take PrEP?

❑ Daily
❑ Event-based (i.e. when you have or think you will have condomless sex)

If you are a daily user, currently taking PrEP:

32a. In the last 2 weeks, how many doses have you missed?

.... (please specify) ❑ None ❑ Don't know

If you are an event-based user, currently taking PrEP:

32b. In the last 4 weeks, were there episodes of condomless anal sex in which you did not use PrEP?

❑ Yes ❑ No ❑ Don't know

33. New forms of PrEP are in development. If all were available and equally effective in preventing HIV, tick which you would you consider using:

❑ Long-acting injectable (e.g. an injection every 2 months)
❑ Removable implant ❑ Adhesive skin patch
❑ None of these

34. How many people do you personally know that are on PrEP?

.... (please specify) ❑ None

35. I feel satisfied with my sex life.

❑ Agree
❑ Neither agree or disagree
❑ Disagree

36. Comments? Thank you.

2022

GMSHS

Gay,
Bisexual,
& other Men
who have sex with men
SEXUAL HEALTH SURVEY

Conducted by:
University College London and UK Health Security Agency

Funded by:
UK Health Security Agency
University College London
Gilead Sciences Ltd.
Healthy London Partnership (the Fast Track Cities initiative)

This questionnaire is anonymous and confidential

For official use:

Venue: ____________

Date: ____________

Interviewer: ____________
