# Supplementary material for: HIV PrEP use and unmet need among gay, bisexual and other men who have sex with men in London: An analysis of community cross‐sectional surveys in England 2019–2022
Source: HIV Med. 2025 Nov 30;27(3):420–32. doi: 10.1111/hiv.70157 (PMC12968497; doi:10.1111/hiv.70157)
Supplement: Supplementary file 2 — Supplementary S2 Sociodemographic characteristics, service engagement and outcomes, sexual risk and prevention behaviours in GBMSM by self‐perceived HIV status and current PrEP use, 2019 survey iteration. [file HIV-27-420-s001.docx]

Table 1. Sociodemographic characteristics, service engagement and outcomes, sexual risk and prevention behaviours in a) GBMSM by self-perceived HIV status and b) GBMSM providing information on current PrEP use, November 2022 to February 2023

|  | **a) GBMSM by self-perceived HIV status^[[1]](#footnote-1),^^[[2]](#footnote-2)^** | | | **b) GBMSM providing information on current PrEP use^1,2,^^[[3]](#footnote-3)^** | | |
| --- | --- | --- | --- | --- | --- | --- |
|  | **GBMSM participants** | **HIV negative/unknown** | **HIV positive** | **GBMSM participants** | **Current PrEP use** | **No current PrEP use** |
|  | *n*=1090 | *n*=1019 | *n*=71 | *n*=814 | *n*=360 | *n*=454 |
| Recruitment location | | | | | | |
| Bar/pub | 80.3% (875/1090) | 80.4% (819/1019) | 78.9% (56/71) | 80.3% (654/814) | 79.7% (287/360) | 80.8% (367/454) |
| Club | 18.4% (201/1090) | 18.7% (190/1019) | 15.5% (11/71) | 18.8% (153/814) | 19.4% (70/360) | 18.3% (83/454) |
| Sauna | 1.3% (14/1090) | 1.0% (10/1019) | 5.6% (4/71) | 0.9% (7/814) | 0.8% (3/360) | 0.9% (4/454) |
| Provided oral fluid sample | | | | | | |
| No | 29.5% (321/1090) | 29.9% (305/1019) | 22.5% (16/71) | 29.4.% (239/814) | 24.7% (89/360) | 33.0% (150/454) |
| Yes | 70.6% (769/1090) | 70.1% (714/1019) | 77.5% (55/71) | 70.6% (575/814) | 75.3% (271/360) | 67.0% (304/454) |
| HIV Ab result^[[4]](#footnote-4)^ | | | | | | |
| Indeterminate | 0.8% (6/751) | 0.6% (4/696) | 3.6% (2/55) | 0.7% (4/562) | 0.7% (2/270) | 0.7% (2/292) |
| Negative | 91.7% (689/751) | 98.4% (685/696) | 7.3% (4/55) | 98.2% (552/562) | 98.9% (267/270) | 97.6% (285/292) |
| Positive | 7.5% (56/751) | 1.0% (7/696) | 89.1% (49/55) | 1.1% (6/562) | 0.4% (1/270) | 1.7% (5/292) |
| Sociodemographic characteristics | | | | | | |
| Age | | | | | | |
| Mean [SD] | 39 [11.7] | 39 [11.7] | 43 [11.6] | 38 [11.5] | 37 [10.1] | 39 [12.4] |
| Median [IQR] | 36 [30-47] | 36 [30-46] | 43 [34-52] | 35 [30-45] | 35 [30-42] | 36 [30-48] |
| Age group |  |  |  |  |  |  |
| 18-24 | 6.4% (68/1066) | 6.6% (66/995) | 2.8% (2/71) | 7.4% (59/796) | 5.7% (20/352) | 8.8% (39/444) |
| 25-34 | 37.8% (403/1066) | 38.8% (386/995) | 23.9% (17/71) | 40.1% (319/796) | 44.0% (155/352) | 36.9% (164/444) |
| 35-44 | 26.5% (282/1066) | 26.4% (263/995) | 26.8% (19/71) | 26.6% (212/796) | 29.6% (104/352) | 24.3% (108/444) |
| ≥45 | 29.4% (313/1066) | 28.1% (280/995) | 46.5% (33/71) | 25.9% (206/796) | 20.7% (73/352) | 30.0% (133/444) |
| Ethnic group | | | | | | |
| White | 72.4% (787/1087) | 72.4% (736/1016) | 71.8% (51/71) | 72.4% (587/811) | 72.4% (260/359) | 72.4% (327/452) |
| Black | 3.0% (33/1087) | 3.0% (30/1016) | 4.2% (3/71) | 2.7% (22/811) | 3.1% (11/359) | 2.4% (11/452) |
| South East Asian | 2.7% (29/1087) | 2.8% (28/1016) | 1.4% (1/71) | 2.8% (23/811) | 3.1% (11/359) | 2.7% (12/452) |
| Asian | 4.4% (48/1087) | 4.2% (43/1016) | 7.0% (5/71) | 4.4% (36/811) | 3.9% (14/359) | 4.9% (22/452) |
| Latin American | 9.3% (101/1087) | 9.1% (92/1016) | 12.7% (9/71) | 8.6% (70/811) | 9.2% (33/359) | 8.2% (37/452) |
| Mixed/other | 8.2% (89/1087) | 8.6% (87/1016) | 2.8% (2/71) | 9.0% (73/811) | 8.4% (30/359) | 9.5% (43/452) |
| UK-born | | | | | | |
| No | 44.7% (481/1075) | 44.1% (443/1005) | 54.3% (38/70) | 44.3% (356/803) | 47.2% (167/354) | 42.1% (189/449) |
| Yes | 55.3% (594/1075) | 55.9% (562/1005) | 45.7% (32/70) | 55.7% (447/803) | 52.8% (187/354) | 57.9% (260/449) |
| Residence | | | | | | |
| London | 78.1% (850/1089) | 77.7% (791/1018) | 83.1% (59/71) | 79.3% (645/813) | 83.3% (300/360) | 76.2% (345/453) |
| Outside London | 12.8% (139/1089) | 13.0% (132/1018) | 9.9% (7/71) | 11.8% (96/813) | 9.7% (35/360) | 13.5% (61/453) |
| Outside UK | 9.2% (100/1089) | 9.3% (95/1018) | 7.0% (5/71) | 8.9% (72/813) | 6.9% (25/360) | 10.4% (47/453) |
| Current employment | | | | | | |
| No | 10.2% (108/1063) | 10.0% (99/994) | 13.0% (9/69) | 9.3% (74/794) | 7.9% (28/353) | 10.4% (46/441) |
| Yes | 89.8% (955/1063) | 90.0% (895/994) | 87.0% (60/69) | 90.7% (720/794) | 92.1% (325/353) | 89.6% (395/441) |
| Education since age 16 | | | | | | |
| 0-2 years | 14.7% (157/1071) | 14.7% (147/1001) | 14.3% (10/70) | 14.3% (115/803) | 13.5% (48/356) | 15.0% (67/447) |
| ≥ 2 years/still full-time | 85.3% (914/1071) | 85.3% (854/1001) | 85.7% (60/70) | 85.7% (688/803) | 86.5% (308/356) | 85.0% (380/447) |
| Service engagement and outcomes | | | | | | |
| SHS visit in the last year | | | | | | |
| No | 34.0% (363/1068) | 35.8% (357/997) | 8.5% (6/71) | 29.4% (235/800) | 2.8% (10/355) | 50.6% (225/445) |
| Yes | 66.0% (705/1068) | 64.2% (640/997) | 91.6% (65/71) | 70.6% (565/800) | 97.2% (345/355) | 49.4% (220/445) |
| Last HIV test | | | | | | |
| Last 3 months | 40.5% (422/1041) | 40.9% (400/979) | 35.5% (22/62) | 46.5% (367/789) | 78.1% (274/351) | 21.2% (93/438) |
| Between 3-12 months | 25.0% (260/1041) | 25.8% (253/979) | 11.3% (7/62) | 26.7% (211/789) | 19.4% (68/351) | 32.7% (143/438) |
| More than a year ago | 18.9% (197/1041) | 19.8% (194/979) | 4.8% (3/62) | 16.6% (131/789) | 2.3% (8/351) | 28.1% (123/438) |
| Over 5 years ago | 10.4% (108/1041) | 8.0% (78/979) | 48.4% (30/62) | 5.1% (40/789) | 0.0% (0/351) | 9.1% (40/438) |
| Never | 5.2% (54/1041) | 5.5% (54/979) | 0.0% (0/62) | 5.1% (40/789) | 0.3% (1/351) | 8.9% (39/438) |
| ≥ 2 HIV tests in the last year | | | | | | |
| No | 52.0% (536/1031) | 51.3% (501/976) | 63.6% (35/55) | 44.3% (348/786) | 11.5% (40/349) | 70.5% (308/437) |
| Yes | 48.0% (495/1031) | 48.7% (475/976) | 36.4% (20/55) | 55.7% (438/786) | 88.5% (309/349) | 29.5% (129/437) |
| ≥ 4 STI tests in the last year | | | | | | |
| No | 83.6% (869/1040) | 83.6% (811/970) | 82.9% (58/70) | 81.0% (632/780) | 64.2% (224/349) | 94.7% (408/431) |
| Yes | 16.4% (171/1040) | 16.4% (159/970) | 17.1% (12/70) | 19.0% (148/780) | 35.8% (125/349) | 5.3% (23/431) |
| STI diagnosis in the last year | | | | | | |
| No | 73.9% (766/1037) | 75.7% (733/968) | 47.8% (33/69) | 71.9% (562/782) | 53.3% (188/353) | 87.2% (374/429) |
| Yes | 26.1% (271/1037) | 24.3% (235/968) | 52.2% (36/69) | 28.1% (220/782) | 46.7% (165/353) | 12.8% (55/429) |
| Sexual risk and prevention behaviours | | | | | | |
| CAS in the last 3 months | | | | | | |
| No | 41.4% (437/1056) | 42.0% (415/988) | 32.4% (22/68) | 39.3% (313/797) | 23.0% (81/353) | 52.3% (232/444) |
| Yes | 58.6% (619/1056) | 58.0% (573/988) | 67.7% (46/68) | 60.7% (484/797) | 77.1% (272/353) | 47.8% (212/444) |
| ≥ 5 CAS partners in the last year | | | | | | |
| No | 71.2% (669/940) | 72.8% (638/877) | 49.2% (31/63) | 69.0% (501/726) | 40.4% (130/322) | 91.8% (371/404) |
| Yes | 28.8% (271/940) | 27.3% (239/877) | 50.8% (32/63) | 31.0% (225/726) | 59.6% (192/322) | 8.2% (33/404) |
| Chemsex in the last year^[[5]](#footnote-5)^ | | | | | | |
| No | 83.9% (836/997) | 85.2% (795/933) | 64.1% (41/64) | 82.7% (623/753) | 75.8% (257/339) | 88.4% (366/414) |
| Yes | 16.2% (161/997) | 14.8% (138/933) | 35.9% (23/64) | 17.3% (130/753) | 24.2% (82/339) | 11.6% (48/414) |
| HIV PEP in the last year | | | | | | |
| No | 90.7% (989/1090) | 91.0% (927/1019) | 87.3% (62/71) | 89.1% (725/814) | 81.7% (294/360) | 94.9% (431/454) |
| Yes | 9.3% (101/1090) | 9.0% (92/1019) | 12.7% (9/71) | 10.9% (89/814) | 18.3% (66/360) | 5.01% (23/454) |
| DoxyPEP^[[6]](#footnote-6)^ | | | | | | |
| No | 85.4% (860/1007) | 86.6% (817/943) | 67.2% (43/64) | 84.0% (637/758) | 72.0% (239/332) | 93.4% (398/426) |
| Yes | 14.6% (147/1007) | 13.4% (126/943) | 32.8% (21/64) | 16.0% (121/758) | 28.0% (93/332) | 6.6% (28/426) |
| PrEP use in the last year | | | | | | |
| No | - | 53.7% (539/1004) | - | 43.9% (357/813) | 0.0% (0/360) | 78.8% (357/453) |
| Yes | - | 46.3% (465/1004) | - | 56.1% (456/813) | 100% (360/360) | 21.2% (96/453) |
| Current PrEP use | | | | | | |
| No | - | 55.8% (454/814) | - | - | - | - |
| Yes | - | 44.2% (360/814) | - | - | - | - |

Table 2. Sociodemographic characteristics, service engagement and outcomes, sexual risk and prevention behaviours in a) GBMSM by self-perceived HIV status and b) GBMSM providing information on current PrEP use, June to August 2019

|  | **a) GBMSM by self-perceived HIV status^[[7]](#footnote-7),^^[[8]](#footnote-8)^** | | | **b) GBMSM providing information on current PrEP use^1,2,^^[[9]](#footnote-9)^** | | |
| --- | --- | --- | --- | --- | --- | --- |
|  | **GBMSM participants** | **HIV negative/unknown** | **HIV positive** | **GBMSM participants** | **Current PrEP use** | **No current PrEP use** |
|  | *n*=1408 | *n*=1288 | *n*=120 | *n*=1233 | *n*=245 | *n*=988 |
| Recruitment location | | | | | | |
| Bar/pub | 72.9% (1026/1408) | 72.6% (935/1288) | 75.8% (91/120) | 72.4% (893/1233) | 70.6% (173/245) | 72.9% (720/988) |
| Club | 22.7% (320/1408) | 23.1% (298/1288) | 18.3% (22/120) | 23.2% (286/1233) | 24.1% (59/245) | 23.0% (227/988) |
| Sauna | 4.4% (62/1408) | 4.3% (55/1288) | 5.8% (7/120) | 4.4% (54/1233) | 5.3% (13/245) | 4.2% (41/988) |
| Provided oral fluid sample | | | | | | |
| No | 38.3% (539/1408) | 38.3% (493/1288) | 38.3% (46/120) | 37.5% (462/1233) | 35.1% (86/245) | 38.1% (376/988) |
| Yes | 61.7% (869/1408) | 61.7% (795/1288) | 61.7% (74/120) | 62.5% (771/1233) | 64.9% (159/245) | 61.9% (612/988) |
| HIV Ab result^[[10]](#footnote-10)^ | | | | | | |
| Indeterminate | 0.5% (4/855) | 0.1% (1/784) | 4.2% (3/71) | 0.1% (1/760) | 0.7% (1/155) | 0.0% (0/605) |
| Negative | 91.2% (780/855) | 99.1% (777/784) | 4.2% (3/71) | 99.1% (753/760) | 97.4% (151/155) | 99.5% (602/605) |
| Positive | 8.3% (71/855) | 0.8% (6/784) | 91.6% (65/71) | 0.8% (6/760) | 2.0% (3/155) | 0.5% (3/605) |
| Sociodemographic characteristics | | | | | | |
| Age | | | | | | |
| Mean [SD] | 37 [11.2] | 37 [11.1] | 44 [10.0] | 37 [11.1] | 35 [9.2] | 37 [11.5] |
| Median [IQR] | 35 [29–45] | 35 [28–44] | 45 [37–51] | 35 [28–44] | 33 [28–41] | 35 [29–45] |
| Age group |  |  |  |  |  |  |
| 18-24 | 10.0% (139/1392) | 10.8% (138/1276) | 0.9% (1/116) | 10.8% (132/1223) | 8.6% (21/244) | 11.3% (111/979) |
| 25-34 | 37.6% (523/1392) | 39.1% (499/1276) | 20.7% (24/116) | 38.5% (471/1223) | 43.9% (107/244) | 37.2% (364/979) |
| 35-44 | 27.4% (381/1392) | 27.3% (348/1276) | 28.5% (33/116) | 27.6% (337/1223) | 35.3% (86/244) | 25.6% (251/979) |
| ≥45 | 25.1% (349/1392) | 22.8% (291/1276) | 50.0% (58/116) | 23.1% (283/1223) | 12.3% (30/244) | 25.8% (253/979) |
| Ethnic group | | | | | | |
| White | 75.5% (1062/1406) | 75.5% (971/1286) | 75.8% (91/120) | 75.6% (930/1231) | 72.2% (177/245) | 76.4% (753/986) |
| Black | 4.1% (57/1406) | 3.9% (50/1286) | 5.8% (7/120) | 3.9% (48/1231) | 3.3% (8/245) | 4.1% (40/986) |
| South East Asian | 2.2% (31/1406) | 2.4% (31/1286) | 0.0% (0/120) | 2.3% (28/1231) | 2.9% (7/245) | 2.1% (21/986) |
| Asian | 4.3% (61/1406) | 4.6% (59/1286) | 1.7% (2/120) | 4.6% (57/1231) | 5.3% (13/245) | 4.5% (44/986) |
| Latin American | 4.9% (69/1406) | 4.4% (57/1286) | 10.0% (12/120) | 4.5% (55/1231) | 5.3% (13/245) | 4.3% (42/986) |
| Mixed/other | 9.0% (126/1406) | 9.2% (118/1286) | 6.7% (8/120) | 9.2% (113/1231) | 11.0% (27/245) | 8.7% (86/986) |
| UK-born | | | | | | |
| No | 46.3% (642/1386) | 46.4% (589/1269) | 45.3% (53/117) | 46.1% (560/1216) | 49.4% (119/241) | 45.2% (441/975) |
| Yes | 53.7% (744/1386) | 53.6% (680/1269) | 54.7% (64/117) | 54.0% (656/1216) | 50.6% (122/241) | 54.8% (534/975) |
| Residence | | | | | | |
| London | 82.0% (1133/1381) | 81.3% (1026/1262) | 89.9% (107/119) | 81.4% (983/1208) | 85.4% (204/239) | 80.4% (779/969) |
| Outside London | 11.7% (162/1381) | 12.1% (153/1262) | 7.6% (9/119) | 12.2% (147/1208) | 8.4% (20/239) | 13.1% (127/969) |
| Outside UK | 6.2% (86/1381) | 6.6% (83/1262) | 2.5% (3/119) | 6.5% (78/1208) | 6.3% (15/239) | 6.5% (63/969) |
| Current employment | | | | | | |
| No | 12.6% (175/1393) | 12.2% (155/1274) | 16.8% (20/119) | 12.0% (146/1221) | 9.9% (24/243) | 12.5% (122/978) |
| Yes | 87.4% (1218/1393) | 87.8% (1119/1274) | 83.2% (99/119) | 88.0% (1075/1221) | 90.1% (219/243) | 87.5% (856/978) |
| Education since age 16 | | | | | | |
| 0-2 years | 16.1% (222/1377) | 15.6% (196/1260) | 22.2% (26/117) | 15.6% (188/1208) | 11.2% (27/241) | 16.7% (161/967) |
| ≥ 2 years/still full-time | 83.9% (1155/1377) | 84.4% (1064/1260) | 77.8% (91/117) | 84.4% (1020/1208) | 88.8% (214/241) | 83.4% (806/967) |
| Service engagement and outcomes | | | | | | |
| SHS visit in the last year | | | | | | |
| No | 30.2% (419/1387) | 32.3% (409/1267) | 8.3% (10/120) | 32.5% (395/1217) | 2.9% (7/243) | 39.8% (388/974) |
| Yes | 69.8% (968/1387) | 67.7% (858/1267) | 91.7% (110/120) | 67.5% (822/1217) | 97.1% (236/243) | 60.2% (586/974) |
| Last HIV test | | | | | | |
| Last 3 months | 46.6% (642/1378) | 47.0% (597/1269) | 41.3% (45/109) | 47.5% (578/1217) | 88.1% (214/243) | 37.4% (364/974) |
| Between 3-12 months | 24.6% (339/1378) | 25.7% (326/1269) | 11.9% (13/109) | 25.2% (307/1217) | 10.7% (26/243) | 28.9% (281/974) |
| More than a year ago | 16.9% (233/1378) | 17.8% (226/1269) | 6.4% (7/109) | 17.9% (218/1217) | 0.8% (2/243) | 22.2% (216/974) |
| Over 5 years ago | 7.7% (106/1378) | 5.0% (63/1269) | 39.5% (43/109) | 4.9% (60/1217) | 0.4% (1/243) | 6.1% (59/974) |
| Never | 4.2% (58/1378) | 4.5% (57/1269) | 0.9% (1/109) | 4.4% (54/1217) | 0.0% (0/243) | 5.5% (54/974) |
| ≥ 2 HIV tests in the last year | | | | | | |
| No | 46.1% (575/1248) | 45.2% (528/1168) | 58.8% (47/80) | 45.0% (503/1119) | 6.7% (16/239) | 55.3% (487/880) |
| Yes | 53.9% (673/1248) | 54.8% (640/1168) | 41.3% (33/80) | 55.1% (616/1119) | 93.3% (223/239) | 44.7% (393/880) |
| ≥ 4 STI tests in the last year | | | | | | |
| No | 82.4% (1053/1278) | 82.0% (956/1166) | 86.6% (97/112) | 81.8% (921/1126) | 50.6% (119/235) | 90.0% (802/891) |
| Yes | 17.6% (225/1278) | 18.0% (210/1166) | 13.4% (15/112) | 18.2% (205/1126) | 49.4% (116/235) | 10.0% (89/891) |
| STI diagnosis in the last year | | | | | | |
| No | 75.4% (1036/1375) | 76.4% (960/1256) | 63.9% (76/119) | 76.4% (922/1207) | 41.0% (100/244) | 85.4% (822/963) |
| Yes | 24.7% (339/1375) | 23.6% (296/1256) | 36.1% (43/119) | 23.6% (285/1207) | 59.0% (144/244) | 14.6% (141/963) |
| Sexual risk and prevention behaviours | | | | | | |
| CAS in the last 3 months | | | | | | |
| No | 48.5% (668/1378) | 49.5% (625/1262) | 37.1% (43/116) | 49.6% (603/1216) | 16.9% (41/243) | 57.8% (562/973) |
| Yes | 51.5% (710/1378) | 50.5% (637/1262) | 62.9% (73/116) | 50.4% (613/1216) | 83.1% (202/243) | 42.2% (411/973) |
| ≥ 5 CAS partners in the last year | | | | | | |
| No | 77.9% (968/1242) | 79.4% (904/1138) | 61.5% (64/104) | 79.1% (867/1096) | 39.4% (93/236) | 90.0% (774/860) |
| Yes | 22.1% (274/1242) | 20.6% (234/1138) | 38.5% (40/104) | 20.9% (229/1096) | 60.6% (143/236) | 10.0% (86/860) |
| Chemsex in the last year^[[11]](#footnote-11)^ | | | | | | |
| No | 82.5% (1105/1340) | 83.7% (1025/1225) | 69.6% (80/115) | 83.8% (990/1182) | 66.0% (155/235) | 88.2% (835/947) |
| Yes | 17.5% (235/1340) | 16.3% (200/1225) | 30.4% (35/115) | 16.2% (192/1182) | 34.0% (80/235) | 11.8% (112/947) |
| HIV PEP in the last year | | | | | | |
| No | 93.3% (1314/1408) | 93.1% (1199/1288) | 95.8% (115/120) | 93.2% (1149/1233) | 84.1% (206/245) | 95.5% (943/988) |
| Yes | 6.7% (94/1408) | 6.9% (89/1288) | 4.2% (5/120) | 6.8% (84/1233) | 15.9% (39/245) | 4.6% (45/988) |
| DoxyPEP^[[12]](#footnote-12)^ | | | | | | |
| No | 94.2% (1257/1335) | 94.9% (1154/1216) | 86.6% (103/119) | 95.0% (1109/1168) | 87.3% (207/237) | 96.9% (902/931) |
| Yes | 5.8% (78/1335) | 5.1% (62/1216) | 13.5% (16/119) | 5.1% (59/1168) | 12.7% (30/237) | 3.1% (29/931) |
| PrEP use in the last year | | | | | | |
| No | - | 75.5% (925/1225) | - | 75.5% (923/1223) | 0.0% (0/245) | 94.4% (923/978) |
| Yes | - | 24.5% (300/1225) | - | 24.5% (300/1223) | 100% (245/245) | 5.6% (55/978) |
| Current PrEP use | | | | | | |
| No | - | 80.1% (988/1233) | - | - | - | - |
| Yes | - | 19.9% (245/1233) | - | - | - | - |

Table 3. Service engagement and outcomes, and sexual risk and prevention behaviours associated with self-reported current PrEP use in self-perceived HIV-negative/unknown GBMSM with identified PrEP-need, June to August 2019

|  | **GBMSM with identified PrEP-need** | | **OR (95% CI)** | ***p* value** | **aOR^[[13]](#footnote-13)^ (95% CI)** | ***p* value** |
| --- | --- | --- | --- | --- | --- | --- |
|  | **Current PrEP use**  ***n*=204** | **No current PrEP use**  ***n*=431** |  |  |  |  |
| Service engagement and outcomes | | | | | | |
| SHS visit in the last year | | | | | | |
| No | 2.0% (4/203) | 34.2% (147/430) | 1 (ref) | <0.001 | 1 (ref) | <0.001 |
| Yes | 98.0% (199/203) | 65.8% (283/430) | 25.84 (9.42-70.93) |  | 25.79 (9.36-71.04) |  |
| ≥ 2 HIV tests in the last year | | | | | | |
| No | 6.4% (13/203) | 49.8% (201/404) | 1 (ref) | <0.001 | 1 (ref) | <0.001 |
| Yes | 93.6% (190/203) | 50.3% (203/404) | 14.47 (7.98-26.23) |  | 15.16 (8.30-27.70) |  |
| Location of last HIV test | | | | | | |
| SHS | 92.1% (187/203) | 70.2% (290/413) | 1 (ref) | <0.001 | 1 (ref) | <0.001 |
| Self-sampling/ testing | 4.4% (9/203) | 14.0% (58/413) | 0.24 (0.12-0.50) |  | 0.24 (0.12-0.50) |  |
| Other | 3.5% (7/203) | 15.7% (65/413) | 0.17 (0.07-0.37) |  | 0.17 (0.08-0.39) |  |
| STI diagnosis in the last year | | | | | | |
| No | 35.8% (73/204) | 80.8% (344/426) | 1 (ref) | <0.001 | 1 (ref) | <0.001 |
| Yes | 64.2% (131/204) | 19.3% (82/426) | 7.53 (5.18-10.94) |  | 8.19 (5.55-12.10) |  |
| Sexual risk and prevention behaviours | | | | | | |
| ≥ 5 CAS partners in the last year | | | | | | |
| No | 31.1% (61/196) | 80.2% (308/384) | 1 (ref) | <0.001 | 1 (ref) | <0.001 |
| Yes | 68.9% (135/196) | 19.8% (76/384) | 8.97 (6.05-13.29) |  | 10.13 (6.69-15.33) |  |
| ≥ 2 casual CAS partners in the last year | | | | | | |
| No | 24.2% (46/190) | 73.0% (267/366) | 1 (ref) | <0.001 | 1 (ref) | <0.001 |
| Yes | 75.8% (144/190) | 27.1% (99/366) | 8.44 (5.64-12.65) |  | 9.21 (6.07-13.99) |  |
| Chemsex in the last year | | | | | | |
| No | 62.9% (124/197) | 82.1% (339/413) | 1 (ref) | 0.007 | 1 (ref) | 0.007 |
| Yes | 37.1% (73/197) | 17.9% (74/413) | 2.70 (1.84-3.96) |  | 2.83 (1.91-4.20) |  |
| DoxyPEP | | | | | | |
| No | 86.5% (173/200) | 96.4% (397/412) | 1 (ref) | <0.001 | 1 (ref) | <0.001 |
| Yes | 13.5% (27/200) | 3.6% (15/412) | 4.13 (2.14-7.96) |  | 4.38 (2.24-8.57) |  |
| HIV PEP use in the last year | | | | | | |
| No | 83.3% (170/204) | 93.3% (402/431) | 1 (ref) | <0.001 | 1 (ref) | <0.001 |
| Yes | 16.7% (34/204) | 6.7% (29/431) | 2.77 (1.64-4.70) |  | 2.61 (1.52-4.47) |  |

1. Self-identified men, including trans men, who self-reported as gay or bisexual, or who had sex with a man in the last year and did not previously participate in the survey in the last three months [↑](#footnote-ref-1)
2. Based on self-perceived HIV status; where self-reported HIV status not specified, based on report of last HIV test as positive or antiretroviral medication use [↑](#footnote-ref-2)
3. Self-perceived HIV-negative/unknown GBMSM [↑](#footnote-ref-3)
4. Where IgG>0.200 [↑](#footnote-ref-4)
5. Chemsex defined as self-reported use of ketamine, gamma hydroxybutyrate(GHB)/gamma butyrolactone(GBL), mephedrone and/or meth amphetamine before or during sex [↑](#footnote-ref-5)
6. Doxycycline post-exposure prophylaxis (doxy PEP) to prevent bacterial STIs [↑](#footnote-ref-6)
7. Self-identified men, including trans men, who self-reported as gay or bisexual, or who had sex with a man in the last year and did not previously participate in the survey in the last three months [↑](#footnote-ref-7)
8. Based on self-perceived HIV status; where self-reported HIV status not specified, based on report of last HIV test as positive or antiretroviral medication use [↑](#footnote-ref-8)
9. Self-perceived HIV-negative/unknown GBMSM [↑](#footnote-ref-9)
10. Where IgG>0.200 [↑](#footnote-ref-10)
11. Chemsex defined as self-reported use of ketamine, gamma hydroxybutyrate(GHB)/gamma butyrolactone(GBL), mephedrone and/or meth amphetamine before or during sex [↑](#footnote-ref-11)
12. Doxycycline post-exposure prophylaxis (doxy PEP) to prevent bacterial STIs [↑](#footnote-ref-12)
13. Adjusted for age-group (employment not used as not significant in multivariate model of sociodemographic characteristics) [↑](#footnote-ref-13)
